# The impact of bariatric surgery on breathing-related polysomnography parameters—Updated systematic review and meta-analysis

**DOI:** 10.3389/frsle.2023.1212936

**Published:** 2023-08-03

**Authors:** Saif Mashaqi, Anas Rihawi, Pooja Rangan, Katherine Ho, Mateen Khokhar, Sonia Helmick, Yazan Ashouri, Daniel Combs, Iman Ghaderi, Sairam Parthasarathy

**Affiliations:** ^1^Department of Pulmonary, Critical Care, Allergy and Sleep Medicine, University of Arizona College of Medicine, Tucson, AZ, United States; ^2^Center for Sleep and Circadian Sciences, University of Arizona Health Sciences, Tucson, AZ, United States; ^3^Division of Pulmonary and Critical Care Medicine, Department of Medicine, Oregon Health and Science University, Portland, OR, United States; ^4^Department of Internal Medicine, The University of Arizona College of Medicine Phoenix, Phoenix, AZ, United States; ^5^Department of Medicine, Banner University Medical Center Phoenix, Phoenix, AZ, United States; ^6^Department of General Surgery, Johns Hopkins University School of Medicine, Baltimore, MD, United States; ^7^Department of Internal Medicine, Dignity Health Medical Group St. Joseph's, Phoenix, AZ, United States; ^8^Department of Pulmonary and Sleep Medicine, Tucson VA Medical Center, Tucson, AZ, United States; ^9^Department of Neurology, St. Vincent Medical Center, Toledo, OH, United States; ^10^Section of Minimally Invasive, Robotic and Bariatric Surgery, Department of Surgery, University of Arizona College of Medicine, Tucson, AZ, United States

**Keywords:** bariatric surgery, body mass index, apnea-hypopnea index, oxygen desaturation index, total sleep time < 90%, mean SpO_2_, nadir SpO_2_, obesity

## Abstract

**Introduction:**

We conducted this systematic review and meta-analysis (SRMA) to evaluate the impact of bariatric surgery on obstructive sleep apnea (OSA) as represented by the following polysomnography (PSG) parameters: apnea-hypopnea index (AHI), oxygen desaturation index (ODI), mean oxygen desaturation (mean SpO_2_), total sleep time spent with SpO_2_ < 90% (T-90), and the nadir of oxygen saturation (L SpO_2_).

**Methods:**

A comprehensive search of the literature was conducted in Ovid MEDLINE, Embase, and Scopus databases from inception to March 31, 2023. Only articles written in English were reviewed. The analysis of all outcomes was performed using a random-effects model. We included 30 studies (two randomized controlled trials and 28 observational studies) in the final quantitative synthesis with a total of 1,369 patients.

**Results:**

We concluded that bariatric surgery (regardless of the type) was associated with reduction in AHI [MD 23.2 events/h (95%CI 19.7, 26.8)], ODI [MD 26.8 events/h (95%CI 21.6, 32.1)], mean SpO_2_ [MD−1.94% (95%CI −2.5, −1.4)], T-90 [MD 7.5min (95%CI 5.0, 10.0)], and L SpO_2_ [MD 9.0% (95%CI −11.8, −6.3)].

**Conclusion:**

Our SRMA results are updates to previously published results and continue to support the positive impact of bariatric surgery on OSA and sleep-related hypoxia.

## 1. Introduction

### 1.1. Rationale

Obstructive sleep apnea (OSA) is the most common sleep-disordered breathing, with a prevalence of 9–38% (Senaratna et al., 2017). One of the main risk factors for OSA is obesity (Tuomilehto et al., 2013). Obesity is a global epidemic with a steady increase in the incidence rates in recent years. Its incidence has tripled since 1975 according to the World Health Organization (WHO) (The Lancet Gastroenterology Hepatology, 2021, Congdon and Amugsi, 2022). The bidirectional relationship between OSA and obesity is complex. An increase in body mass index (BMI) and fat mass contributes to the deposition of fat tissues in the upper airway, including fat pads, uvula, and the base of the tongue. This deposition leads to an upper airway crowdedness, predisposing it to repetitive episodes of collapse during sleep (Turnbull et al., 2018; Yanari et al., 2022). Alternatively, intermittent hypoxia and sleep fragmentation can increase visceral adipose tissue (Harada et al., 2014; Zheng et al., 2022). It is estimated that 40% of people with obesity have notable OSA that warrants treatment (Wolk et al., 2003). Both OSA and obesity are independent risk factors for many co-morbid conditions (such as cardiovascular, cerebrovascular, metabolic, and even neoplastic diseases) (Wolk et al., 2003).

Positive airway pressure (PAP) therapy is considered the treatment of choice for OSA (Epstein et al., 2009). However, adherence to PAP therapy can range between 34 and 50% (Roecklein et al., 2010; Rotenberg et al., 2016). Furthermore, there is evidence of age and sex disparities in PAP therapy adherence that can range between 17 and 71% (Patel et al., 2021). Therefore, alternative therapies are available to patients with poor adherence to PAP therapy. One of these options is weight loss, either conservatively via lifestyle modification or surgically via bariatric surgery. The American Academy of Sleep Medicine (AASM) strongly recommends surgical consultation for bariatric surgery in all patients with OSA and class II/III obesity (BMI ≥ 35 kg/m^2^) (Kent et al., 2021). Similarly, the National Institute of Health (NIH) recommends referral to bariatric surgery in patients with morbid obesity and OSA regardless of PAP compliance (NHLBI, 2000; Kent et al., 2021). Weight loss surgery usually reduces the severity of OSA; however, a complete resolution is unusual (Greenburg et al., 2009; Wong et al., 2018).

Recently, two systematic reviews and meta-analyses (SRMA) were published to synthesize the evidence related to bariatric surgery's impact on OSA (Wong et al., 2018; Zhang et al., 2019). Given the publication of several additional studies since the last SRMA in 2019, we conducted and updated a systematic review of the literature for the quality of the evidence and then conducted a meta-analysis to synthesize the evidence to date.

### 1.2. Objectives

The aim of this systematic review and meta-analysis is to address the following question:

1. Is bariatric surgery [Roux-en-Y gastric bypass (RYGB), sleeve gastrectomy (SG), or adjustable gastric banding (LAGB)] associated with improvement in breathing-related polysomnography parameters [i.e., reduction in apnea-hypopnea index (AHI), reduction in oxygen desaturation index (ODI), increase in mean oxygen saturation (mean SpO_2_), reduction in time spent with SpO_2_ < 90% (T-90), and increase in nadir of oxygen saturation (L SpO_2_)]?

## 2. Methods

The present systematic review and meta-analysis were conducted in accordance with recommendations from the Cochrane Collaboration and are reported in accordance with the Preferred Reporting Items for Systematic Reviews and Meta-Analyses (PRISMA) guidelines.

### 2.1. Eligibility criteria

Studies were selected according to the inclusion criteria outlined below:

i) Human studies that included adults ≥ 18 years old who had undergone bariatric surgery.
ii) Studies that included polysomnography (PSG) or home sleep apnea testing (HSAT) before and after bariatric surgery.
iii) Studies that included any of the following polysomnographic parameters before and after bariatric surgery: apnea-hypopnea index (AHI), oxygen desaturation index (ODI), mean oxygen saturation (mean SpO_2_), time spent with SpO_2_ < 90% (T-90), and nadir of oxygen saturation (L SpO_2_).
iv) Studies that included BMI before and after bariatric surgery.
Only clinical trials (randomized, case-control, cross-sectional, and cohort) were included, but case reports, conference abstracts, review articles (narrative and systematic), and editorials were excluded.
v) Only studies written in English.

Since the goal of this systematic review and meta-analysis is to study the impact of bariatric surgery on OSA in the absence of other treatment options, all studies using positive airway pressure therapy or non-invasive ventilation after bariatric surgery were excluded.

### 2.2. Search methods and data extraction

**Figure 1:**
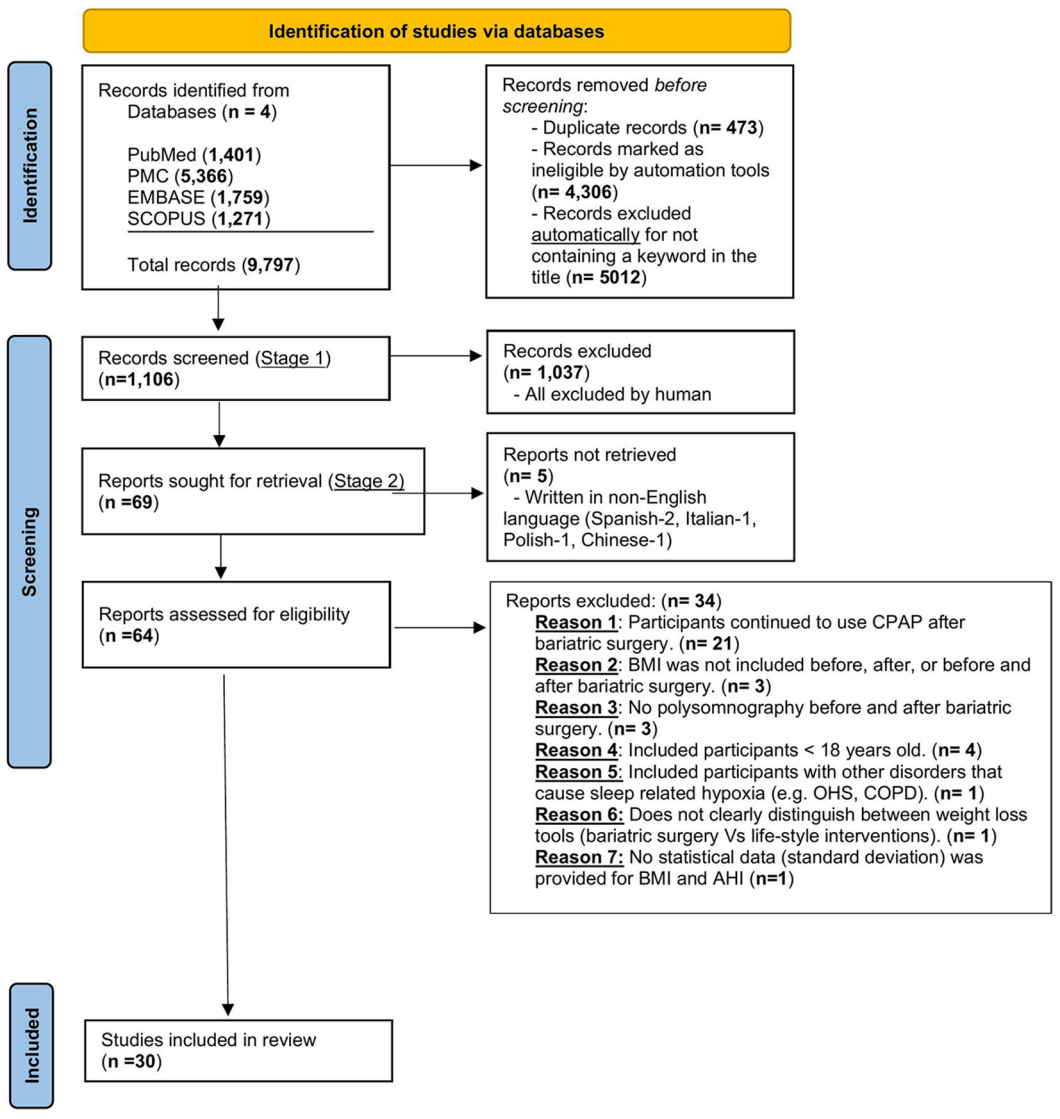
Prisma flow diagram for the identification of appropriate studies for inclusion. OSA, obstructive sleep apnea; CPAP, continuous positive airway pressure therapy; AHI, apnea-hypopnea index; BMI, body mass index; COPD, chronic obstructive pulmonary disease; OHS, obesity hypoventilation syndrome.

The literature search was conducted until 31 March 2023 using the following keywords: “sleep,” “study,” “apnea,” “obstructive,” “bariatric,” “surgery,” “sleeve gastrectomy,” “gastric bypass,” and “gastric banding”. There were no date limits to the search. The search was conducted in Ovid MEDLINE (R); Elsevier Embase (1947–2022); and Elsevier Scopus (1823–2022). Screening was conducted in two stages. In stage one, the authors (SM and AR) independently conducted an initial screening of the titles and abstracts. In stage two, the full text of the records included in stage one of the screening was obtained by authors (SM, DC, MK, SH, PR, and KH) to ensure they met eligibility criteria. Any disagreements were resolved by a discussion between the reviewers. The study selection process is illustrated in Figure 1.

### 2.3. Quality assessment

The quality of included studies was assessed using the following tools:

- Cross-sectional and observational studies using the quality assessment tool for observational cohort and cross-sectional studies developed by the National Heart, Lung, and Blood Institute (Health NIO, 2014).
- Case-control studies using the quality assessment tool for case-control studies developed by the National Heart, Lung, and Blood Institute (2021).
- Randomized control trials (RCTs) using the Cochrane Risk of Bias (ROBINS2) assessment tool (Higgins et al., 2019).

Samples of these quality assessment tools are included as Supplementary Tables S1, S3.

### 2.4. Certainty assessment

Two authors (SM and AR) independently assessed the certainty of the evidence. Five GRADE considerations (study limitations, consistency of effect, imprecision, indirectness, and publication bias) were used to assess the certainty of the body of evidence as they are related to the studies that contributed data to the meta-analyses for the prespecified outcomes. The certainty of evidence was reported as high, moderate, low, or very low. We used the methods and recommendations described in Sections 8.5 and 8.7 and Chapters 11 and 12 of the Cochrane Handbook for Systematic Reviews of Interventions. We justified all decisions to downgrade or upgrade the certainty of studies using footnotes, and we provided comments to aid the reader's understanding of the results where necessary (Mudano et al., 2019).

### 2.5. Result synthesis and statistical analysis

Data were pooled for all studies that examined the effect of surgical weight loss on OSA severity and BMI. The pooled estimate of the mean difference was used as the primary outcome measure and 95% confidence intervals were calculated. A random effects model by DerSimonian and Laird was chosen to synthesize the data throughout because it allows within- and between-study variations, which are applicable to this meta-analysis that includes mostly observational cross-sectional studies with inherently more variability (Dersimonian and Laird, 1986).

Heterogeneity between studies was tested using both the *I*^2^ statistic, where *I*^2^-values of 25, 50, and 75% were defined as mild, moderate, and high heterogeneity, respectively. Publication bias was assessed by visual inspection of funnel plots to examine possible asymmetry and by using the Egger regression asymmetric test (Bowden et al., 2015). To explain the heterogeneity between studies and to examine the influence of various factors, we performed a meta-regression. The following factors were studied: baseline AHI and BMI, age, study design (observational vs. randomized control trial), type of bariatric surgery, the continent where the study was done, study results used more than once, and duration between bariatric surgery and follow-up sleep study. Leave-one-out sensitivity analyses were performed to further explore the changes in our findings by iteratively removing one influential study at a time. All analyses were conducted in STATA software version 17.0 (Stata Corp., College Station, TX). The significance was set at two-tailed *p*-values of 0.05.

## 3. Results

### 3.1. Included studies

A total of 9,797 articles were identified by searching the different databases. Following the removal of duplicate articles (*n* = 473), articles unreadable by automation tools (*n* = 4,306), and articles excluded for other reasons (*n* = 3,912), a total of 1,106 articles were screened using the title and the abstract. The details of the included/excluded articles were added to the PRISMA flow diagram. This yielded 69 articles that were fully reviewed. After applying the inclusion/exclusion criteria, a total of 30 articles were deemed eligible for both qualitative and quantitative (meta-analysis) synthesis. Two of these articles were RCTs (Aguiar et al., 2014; Bakker et al., 2018). All the non-randomized controlled trials except two studies [one case-control (Busetto et al., 2005) and one cross-sectional (Lage-Hansen et al., 2018)] were observational (i.e., 26 studies). Out of these 26 observational studies, 19 were prospective (Pillar et al., 1994; Valencia-Flores et al., 2004; Pallayova et al., 2011; Krieger et al., 2012; Bakker et al., 2013, 2014; Fredheim et al., 2013; Bae et al., 2014; Karaköse et al., 2014; Zou et al., 2015; Del Genio et al., 2016; Jiao et al., 2016; Shaarawy et al., 2016; Xu et al., 2016; Peromaa-Haavisto et al., 2017; Tirado et al., 2017; Al-Jumaily et al., 2018; Chierakul et al., 2020; Yilmaz Kara et al., 2021) and 7 retrospective (Peiser et al., 1984; Fritscher et al., 2007; Morong et al., 2014; Obeidat et al., 2020; Song et al., 2021; Wu et al., 2022; Yanari et al., 2022) in study design. Figure 1, the PRISMA flow diagram, demonstrates the flow of records from the initial search through the selection process, the number of records included, and the reasons for exclusion. Tables 1, 2 illustrate the characteristics of the included studies. The quality assessment of the included studies is shown in Supplementary Tables S2 (“non-RCT” and S4 “RCT”). The quality of evidence across all studies included was very low (Supplementary Table S5). This is related to several factors, including a high risk of bias and publication bias.

**Table 1:** Characteristics of the included studies.

**References**	**Country**	**Study design**	**Bariatric surgery**	Sample size^*^(M)(A) (before)	Sample size^*^(after)	**Follow up (months)**	**BMI kg/m^2^ (before)**	**BMI kg/m^2^ (after)**	**Sleep study (PSG vs. HSAT)**	**Comments**
Lage-Hansen et al. (2018)	Denmark	P (cross-sectional)	- RYGB	24 (13) (44)	24	12	44.4 ± 5.2	30.8 ± 4.8	CRM (HSAT)—Embletta	- Participants had surgery (56). - Participants with OSA (33, 59%). - Declined to continue study (9), not included in analysis.
Al-Jumaily et al. (2018) (1)	USA	P	- RYGB	10 (0) (45)	10	6	48.5 ± 6.5	33.7 ± 4	PSG	−4 patients refused PSG at 12 months.
Al-Jumaily et al. (2018) (2)	USA	P	- RYGB	10 (0) (45)	6	12	48.5 ± 6.5	30.3 ± 3.6	PSG	−4 patients refused PSG at 12 months.
Bakker et al. (2018) (1)	USA	RCT	- GB	−28 (16) (51) ^*^Control (21)	25	9	39.1 ± 2.9	35.9 ± 3.5	PSG	- Control included CPAP therapy. - CPAP was more effective than GB in reducing the “effective AHI” at 9 months.
Bakker et al. (2018) (2)	USA	RCT	- GB	•28 (16) (51) ^*^Control (21)	24	18	39.1 ± 2.9	35.7 ± 3.9	PSG	
Song et al. (2021)	China	R	- RYGB	37 (16) (49)	37	12	31 ± 3.4	24 ± 2.2	PSG	- All participants were diabetic.
Busetto et al. (2005)	USA	P (case-control)	- IGB	17 (17)	17	6	55.8 ± 9.9	48.6 ± 11.2	PSG	- One patient did not tolerate IGB. - The change in AHI significantly correlated with waist size.
Shaarawy et al. (2016)	Kuwait	P (observational)	- SG	27	22 (13) (37)	12	48.2 ± 7.3	35.9 ± 4.8	PSG	- Five dropped out. - Included only severe OSA.
Peiser et al. (1984) (1)	Israel	R	- RYGB	15 (14) (45)	15	3	48 ± 8.6	36.3 ± 7.4	PSG	None
Peiser et al. (1984) (2)	Israel	R	- RYGB	15 (14) (45)	6	5	48 ± 8.6	34 ± 4.0	PSG	None
Obeidat et al. (2020)	Jordan	R	- RYGB	112 (52) (37)	112	30 (mean)	49.8 ± 7.8	33.7 ± 13.6	PSG	−179 patients underwent bariatric surgery but only 112 patients had OSA.
Bakker et al. (2013)	USA	P	- GB - RYGB	27 (2) (43)	27	2	43.7 (42.0, 51.4)	42.7 (30.1, 38.7)	PSG	- Control group were patients treated with CPAP.
Bakker et al. (2014) (1) 6 m	USA	P	- GB - RYGB	12 (2) (43) 15 (CPAP)	12	−6 - 12–18	43.7 (42.0, 51.4)	−32.7 (30.1, 38.7)	PSG	- Total of 27 participants (Bariatric surgery vs. CPAP).
Bakker et al. (2014) (2) 12–18 m	USA	P	- GB - RYGB	12 (2) (43) 15 (CPAP)	12	12–18	43.7 (42.0, 51.4)	−28.3 (25.3, 37.5)	PSG	- Total of 27 participants (Bariatric surgery vs. CPAP).
Morong et al. (2014)	Netherlands	R	Non specified	162 (43) (47)	91	7 (median)	44.8 (40.0–49.6)	35.7 (31.6–40.2)	PSG	- Positional OSA in patients undergoing bariatric surgery is lower than the general population.
Jiao et al. (2016)	China	P	RYGB	39 (15) (48)	39	6–12	30.73 ± 3.66	24.24 ± 2.70	PSG	- Diabetic patients.
Krieger et al. (2012)	USA	P	- GB	30 (10) (44)	24	12	47.18 ± 11.01	35.62 ± 8.23	PSG	- GB improves OSA and leptins.
Pallayova et al. (2011)	USA	P	- RYGB - SG (1) - BPDDS (1)	23 (9) (42)	23	12	52.3 ± 7.4	35.7 ± 6.3	PSG	- sTNFα receptor 2 could be a marker OSA treatment by bariatric surgery.
Del Genio et al. (2016)	Italy	P	- SG	36	36	60	51.3 ± 11.6	32.1 ± 6.6	PSG	- Patients who did not respond to SG were found to have nasal pathology.
Pillar et al. (1994) (1)	Israel	P	- RYGB - VBG	14 (11) (46)	14	4.5	45 ± 7.2	33 ± 7.5	PSG	None
Pillar et al. (1994) (2)	Israel	P	- RYGB - VBG	14 (11) (46)	14	90	45 ± 7.2	35 ± 6.0	PSG	None
Fredheim et al. (2013)	Norway	Non-randomized clinical trial	- RYGB	139 (40) (45)	133	12	47.5 ± 5.6	33.5 ± 3.7	(HSAT)—Embletta	OSA to ILI vs. surgery.
Zou et al. (2015)	China	P	- RYGB	54	44 (18) (48)	10 (mean)	31.1 ± 3.4	24.4 ± 2.6	PSG	None
Aguiar et al. (2014)	Brazil	RCT	- RYGB	16 (3) (40)	16	3	48.2 ± 8.6	36.9 ± 6.7	PSG	−52 patients were allocated to control (36) and surgery (16).
Bae et al. (2014)	Korea	P	- RYGB	67	10 (5) (39)	12	39.9 ± 8.3	26.9 ± 4.4	PSG	- Out of the 67 who had surgery, 61 had OSA.
Fritscher et al. (2007)	Brazil	R (Observational)	RYGB	13 (9) (44.6)	12	24.2 ± 6.4	55.5 ± 10.1	34.1 ± 8.1	PSG	−4 patients were excluded despite having mild OSA. AHI < 15 events per h.
Xu et al. (2016)	China	P	RYGB	39 (15) (46.3, 50.2) in males and females.	35	6	−31.1 (m) (29.7–33.0) - 31.1 (f) (29.5–32.9)	−24.9 (m) (23.9–26.2) - 23.9 (f) (22.7–25.3)	PSG	- Follow up was at 6 and 24 months. However, no PSG at 24 months post-surgery.
Valencia-Flores et al. (2004)	Mexico	P	VBG RYGB D-RYGB	29 (13) (37.9)	29	12	Group 1: 56.5 ± 12.3 Group 2: 54.9 ± 13.1	Group 1: 39.2 ± 8.5 Group 2: 39.2 ± 7.9	PSG	- Two groups (both have OSA before surgery) but in group 1 AHI was less severe compared to group 2. - For mean SPO_2_, took the mean of both REM and NREM
Tirado et al. (2017)	Spain	P (Observational)	RYGB SG	66 (12) (42.3)	66	12	45.6 ± 6.19	29.0 ± 3.69	PSG	None
Karaköse et al. (2014)	Turkey	P	- SG - Mini GB	17 (5) (40)	17	8.35 (mean)	48.48 ± 6.45	34.25 ± 4.86	PSG	Forty patients were enrolled in the study. 33 patients underwent PSG and bariatric surgery. 7 patients did not undergo the control PSG due to symptom being excluded.
Peromaa-Haavisto et al. (2017)	Finland	P	RYGB	132 (-) (50.7)	128	12	43.9 ± 6.4	33.0 ± 5.1	HSAT Embletta	−197 initially but only 132 fulfilled OSA.
Chierakul et al. (2020)	Thailand	P	Not specified (only BS)	24	24 (14) (35)	7.8	51.6 ± 8.7	38.2 ± 6.8	PSG	−96% of participants had Severe OSA.
Yilmaz Kara et al. (2021)	Turkey	P	- LSG	31 (14) (44.1)	31	12	49.8 ± 8.5	33.2 ± 8.2	PSG	- None
Wu et al. (2022) (1)	China	R	- LSG	37 (9) (19–37)	37	3	41.5 ± 6.2	35.5 ± 4.4	PSG	- Indexes were better at 6 m than 3 m. No difference b/w 6 and 12 m. - Between 6 and 12 m, BMI ↓, but AHI and LSpO_2_ stabilized. - EWL% and smoking were independent factors determining the efficacy of LSG against morbid obesity.
Wu et al. (2022) (2)	China	R	- LSG	37 (9) (19–37)	37	6	41.5 ± 6.2	31.7 ± 2.6	PSG	
Wu et al. (2022) (3)	China	R	- LSG	37 (9) (19–37)	37	12	41.5 ± 6.2	26.4 ± 3.9	PSG	
Yanari et al. (2022) (1)	Japan	R	- LSG	56 (33) (46.3)	56	6	42.9 ± 6.6	32.3 ± 4.3	PSG	- Excluded mild OSA since they were mixed with participants with No OSA.
Yanari et al. (2022) (2)	Japan	R	- LSG	56 (33) (46.3)	56	12	42.9 ± 6.6	31.6 ± 5.0	PSG	

^*^Sample size include patients before or after surgery who met the diagnosis of OSA.

(1), (2), (3) Represent the same study at different follow-up intervals.

CRM, cardio-respiratory monitoring; RYGB, Roux-en-Y gastric bypass; P, prospective; HSAT, home sleep apnea test; BMI, body mass index; PSG, polysomnography; OSA, obstructive sleep apnea; AASM, American Academy of Sleep Medicine; M, male; A, age; GB, gastric banding; RCT, randomized controlled studies; R, retrospective; IGB, intra-gastric balloon; LSG, laparoscopic sleeve gastrectomy; BPDDS, Biliopancreatic diversion with duodenal switch; VBG, vertical banded gastroplasty; ILI, intensive life-style intervention; D-RYGB, Distal RYGB; CPAP, continuous positive airway pressure; L SpO_2_, nadir of oxygen saturation; EWL, estimated weight loss; AHI, apnea-hypopnea index; BS, bariatric surgery; m, months; s TNFα, soluble tumor necrosis factor alpha.

**Table 2:** Characteristics of the included studies (continued).

**References**	**AHI (events/h) (before)**	**AHI (events/h) (after)**	**ODI (events/h) (before)**	**ODI (events/h) (after)**	**m SPO_2_ (%) (before)**	**m SPO_2_ (%) (after)**	**T-90 (min) (before)**	**T-90 (min) (after)**	**LSaO_2_ (%) (before)**	**LSaO_2_ (%) (after)**	**Events scoring criteria**
Lage-Hansen et al. (2018)	18.2 ± 14.1	4.5 ± 4.9	25.9 ± 21.2	5.5 ± 5.6	-	-	-	-	-	-	Non-AASM
Al-Jumaily et al. (2018) (1)	38.1 ± 29.4	15.7 ± 15	-	-	-	-	-	-	-	-	AASM
Al-Jumaily et al. (2018) (2)	38.1 ± 29.4	5.6 ± 10.2	-	-	-	-	-	-	-	-	AASM
Bakker et al. (2018) (1)	51.5 ± 23.5	39.3 ± 26.4	-	-	-	-	-	-	-	-	AASM
Bakker et al. (2018) (2)	51.5 ± 23.5	34.1 ± 24.6	-	-	-	-	-	-	-	-	AASM
Song et al. (2021) (Pooja)	21.5 ± 15.4	6.4 ± 7.0	-	-	-	-	-	-	80% (95% CI 76–83%)	87% (95% CI 86–89%)	AASM
Busetto et al. (2005)	59.3 ± 18.1	14.0 ± 12.4	-	-	-	-	-	-	N/A	N/A	Non-AASM
Shaarawy et al. (2016)	55.8 ± 8.3	12.8 ± 11.3	52.6 ± 6.5	10.6 ± 6.3	-	-	-	-	67.2 ± 11.3	92.2 ± 5.3	AASM
Peiser et al. (1984) (1)	82 ± 43.6	−15.3 ± 17.3	-	-	-	-	-	-	-	-	Non-AASM
Peiser et al. (1984) (2)	82 ± 43.6	5.5 ± 9.7	-	-	-	-	-	-	-	-	Non-AASM
Obeidat et al. (2020)	29.5 ± 29.0	6.8 ± 10.8	-	-	-	-	-	-	-	-	AASM
Bakker et al. (2013)	18.1 (16.3, 67.5)	10.5 (5.0, 20.8)	-	-	-	-	-	-	78.0 (72.8, 82.8)	79.0 (74.0, 88.0)	AASM
Bakker et al. (2014)	18.1 (16.3, 67.5)	−10.5 (5.0, 20.8) - 6.5 (1.9, 12.8)	-	-	-	-	10.6 (6.1, 24.6)	−19.3 (3.8, 46.5) - 8.7 (1.8, 17.8)	78.0 (72.8, 82.8)	−79.0 (74.0, 88.0) - 84.0 (79.0, 91.0)	AASM
Morong et al. (2014)	21.2 (11.5–34.9)	6.3 (3.2–12.3)	18.6 (10.6–36.1)	6.3 (3.3–12.2)	-	-	-	-	-	-	AASM
Jiao et al. (2016)	13 ± 23.5	3 ± 7	-	-	-	-	-	-	82 ± (14)	89 ± (6)	Not mentioned
Krieger et al. (2012)	34.2 ± 35	19.0 ± 21.7	-	-	95.15 ± 1.84	95.39 ± 1.65	9.22 ± 17.84	4.45 ± 6.85	80.58 ± 6.90	84.00 ± 7.35	AASM
Pallayova et al. (2011)	32.8 (11.4–75.7)	4.7 (2.0–12.9)	-	-	95.2 (94.1–96.1)	96.3 (95.4–97.5)	-	-	78 (69–84)	86 (80–88)	AASM
Del Genio et al. (2016)	32.8 ± 1.7	5.8 ± 1.2	-	-	-	-	-	-	-	-	AASM
Pillar et al. (1994) (1)	40 ± 29	11 ± 16.4	-	-	-	-	-	-	-	-	AASM - Only Apnea was scored
Pillar et al. (1994) (2)	40 ± 29	24 ± 23	-	-	-	-	-	-	-	-	AASM - Only Apnea was scored
Fredheim et al. (2013)	29.3 ± 24.1	7.7 ± 22.2	30.2 ± 24.6	7.3 ± 22.7	92.8 ± 2.5	95.1 ± 2.4	-	-	75.8 ± (9.4)	84.8 (6.2, 11.8)	AASM
Zou et al. (2015)	22.4 ± 17.8	7.1 ± 9.4	25.4 ± 18.6	6.4 ± 9.0	93.4 ± 2.9	95.5 ± 1.7	8 ± 12.7	1.4 ± 3.2	77.1 ± (11.9)	86.7 ± (6.7)	AASM
Aguiar et al. (2014)	15.6 ± 15.5	6.26 ± 7.6	-	-	93.30 (87–97)	94.3 (86.6–98)	-	-	83.25 (70–94)	85 (70–95)	AASM
Bae et al. (2014)	51 ± 34.2	9.3 ± 12.9	61 ± 34.2	8.6 ± 13.0	93.5 ± 2.2	95.8 ± 1.7	-	-	81.8 ± (6.4)	86.0 ± (6.8)	AASM
Fritscher et al. (2007)	46.5 (33–140)	16 (0.9–87)	N/A	N/A	64.7 ± 13.4	78.7 ± 13.7 s	N/A	N/A	64.7 ± 13.4	78.7 ± 13.7%	AASM
Xu et al. (2016)	Men: 21.7 (15.9–30.3) Female: 21.3 (15.4–28.3)	Men: 6.2 (3.4–15.3) Female: 8.9 (5.3–15.2)	Men: 23.9 (16.8–33.8) Female: 28.5 (20.8–38.3)	Men: 4.6 (2.4–12.5) Female: 8.7 (5.1–15.0)	Men: 93.6 (92.1–94.5) Female: 94.0 (93.1–94.8)	Men: 95.4 (94.3–96.4) Female: 95.7 (95.1–96.2)	-	-	Men: CI% 76.1 (69.6–81.1) Female: CI 77.8 (72.1–82.0)	Men: CI% 87.5 (82.4–89.6) Female: CI 85.9 (82.6–88.7)	AASM
Valencia-Flores et al. (2004)	Group 1: 32.7 ± 37.2 Group 2: 71.9 ± 47.9	Group 1: 1.5 ± 1.2 Group 2: 27.1 ± 25.6	-	-	Group 1: NREM: 82.6 ± 13.5 REM: 77.7 ± 15.8 Group 2: NREM: 76.9 ± 11.0 REM: 65.8 ± 13.5	Group 1: NREM: 91.0 ± 3.4 REM: 89.5 ± 5.7 Group 2: NREM: 87.1 ± 4.3 REM: 84.6 ± 5.4	Group 1: 187.7 ± 128 Group 2: 128.1 ± 95.2	Group 1: 93.9 ± 112 Group 2: 218.5 ± 141	-	-	Non-AASM
Tirado et al. (2017)	33.8 ± 26.1	9.14 ± 9.71	33.1 ± 26.0	7.63 ± 7.35	88.5 ± 9.80	92.1 ± 10.06	9.82 ± 16.4	1.14 ± 3.94	-	-	AASM
Karaköse et al. (2014)	28.41 ± 27.64	13.23 ± 21.36	23.84 ± 22.01	11.19 ± 19.76	-	-	-	-	-	-	AASM
Peromaa-Haavisto et al. (2017)	27.6 ± 24.6	9.9 ± 11.2	-	-	92 ± 2.8	93.3 ± 8.4	-	-	-	-	AASM
Chierakul et al. (2020)	87.6 ± 38.9	28.5 ± 21.5	72.3 ± 38.1	24.4 ± 20.7	-	-	-	-	-	-	AASM
Yilmaz Kara et al. (2021)	36.1 ± 27.1	10.3 ± 11.8	26.7 ± 29.5	7.5 ± 9.9	91.5 ± 3.7	94.0 ± 1.6	24.0 ± 29.3	5.4 ± 7.0	74.3 ± 12.1	79.2 ± 14.7	Not mentioned
Wu et al. (2022) (1)	32.2 ± 5.3	19.3 ± 4.6	-	-	-	-	-	-	74.6 ± 8.9	84.5 ± 5.4	Chinese Thoracic Society
Wu et al. (2022) (2)	32.2 ± 5.3	11.1 ± 3.4	-	-	-	-	-	-	74.6 ± 8.9	90.1 ± 3.6	
Wu et al. (2022) (3)	32.2 ± 5.3	9.1 ± 2.2	-	-	-	-	-	-	74.6 ± 8.9	90.8 ± 3.9	
Yanari et al. (2022)	55.0 ± 23.1	27.0 ± 20.6	55.3 ± 23.7	24.9 ± 19.1	-	-	-	-	71.3 ± 11.4	78.7 ± 8.1	Not mentioned
Yanari et al. (2022)	55.0 ± 23.1	24.3 ± 19.9	55.3 ± 23.7	22.4 ± 18.8	-	-	-	-	71.3 ± 11.4	79.9 ± 8.6	

(1), (2), (3) Represent the same study at different follow-up intervals.

Before, before bariatric surgery; after, after bariatric surgery; L SpO_2_, nadir of oxygen saturation; AHI, apnea-hypopnea index; m SpO_2_, mean oxygen saturation; T-90, total sleep time spent with SpO_2_ < 90%; ODI, oxygen desaturation index; AASM, American Academy of Sleep Medicine; REM, Rapid Eye Movement; CI, confidence interval.

### 3.2. Systematic review

Geographically, out of 30 studies, 14 were conducted in Asia, 7 in North America, 6 in Europe, and 3 in South America. A total of 1,369 participants were included in the analysis. They were middle-aged (the range of mean ages was 35–51 years) and primarily women (66% with a range of 0–100% in each study). Individual study sample sizes ranged from 10 to 162 participants, with most enrolling between 23 and 56 participants. The participants underwent laparoscopic SG (5 studies), RYGB (13 studies), and AGB (3 studies). Seven studies included patients who had more than one type of surgery, and two studies did not specify the type of surgery and mentioned only bariatric surgery. Most studies included patients who underwent standard in-laboratory polysomnography (27 studies), and three studies included patients who underwent HSAT. The following parameters were recorded: AHI, ODI, mean SpO_2_, TST < 90, and L SpO_2_. Of the 30 studies, 22 used the American Academy of Sleep Medicine scoring criteria to score obstructive hypopneas, and three studies did not include details on scoring criteria (Table 2).

### 3.3. Meta-analysis

**Figure 2:**
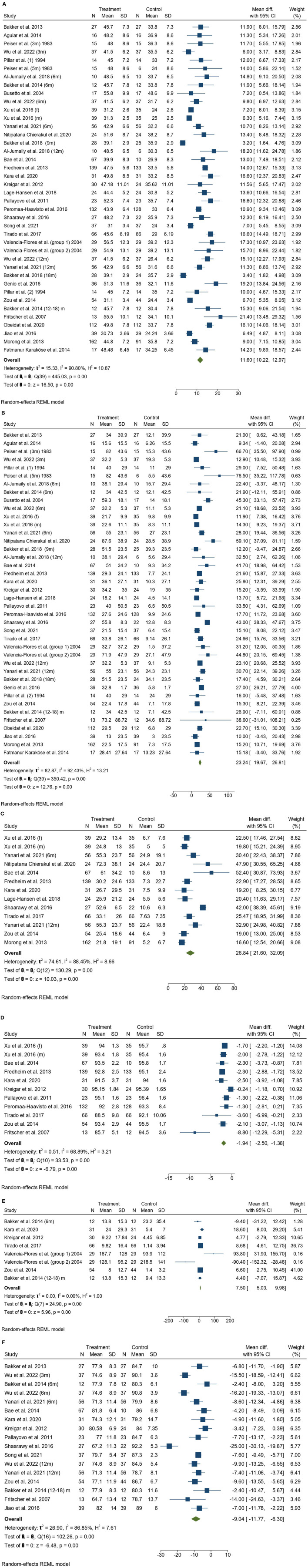
**(A)** A forest plot illustrates body mass index (BMI) before and after bariatric surgery. **(B)** A forest plot illustrates apnea-hypopnea index (AHI) before and after bariatric surgery. **(C)** A forest plot illustrates the oxygen desaturation index (ODI) before and after bariatric surgery. **(D)** A forest plot illustrates mean oxygen desaturation (mean SpO_2_) before and after bariatric surgery. **(E)** A forest plot illustrates the total time spent with SpO_2_ < 90% (T-90) before and after bariatric surgery. **(F)** A forest plot illustrates the nadir of oxygen saturation (L SpO_2_) before and after bariatric surgery. SD, standard deviation; CI, confidence interval.

i) Impact of bariatric surgery on the body mass index (BMI)
Bariatric surgery was associated with a significant reduction in BMI [MD 11.6 kg/m^2^ (95% CI 10.2, 13.0)]. All 30 studies included BMI before and after bariatric surgery (Figure 2A). The BMI reduction was highest immediately following surgery (0–3 months) but subsequently became lower. The studies included in this review measured the BMI at variable intervals post-surgery (e.g., 3 months, 6 months, 9 months, 12 months, 24 months, and 5 years). The mean BMIs before and after bariatric surgery were 45.0 ± 6.9 kg/m^2^ and 32.9 ± 4.9 kg/m^2^, respectively.
ii) Impact of bariatric surgery on the apnea-hypopnea index (AHI)
Bariatric surgery was associated with a significant reduction in the AHI [MD 23.2 events/h (95%CI 19.7, 26.8)]. All 30 studies included AHI before and after bariatric surgery (Figure 2B). The greatest reduction in AHI tended to be in those with the highest baseline AHI (before surgery) with a long follow-up (at least 6 months) (Peiser et al., 1984; Valencia-Flores et al., 2004; Busetto et al., 2005; Bae et al., 2014; Bakker et al., 2018). The mean AHIs before and after bariatric surgery were 40.3 ± 18.7 events/h and 13.5 ± 9.3 events/h, respectively.
iii) Impact of bariatric surgery on the oxygen desaturation index (ODI)
Bariatric surgery was associated with a significant reduction in the ODI [MD 26.8 events/h (95%CI 21.6, 32.1)]. Out of the 30 studies, 11 included ODI before and after bariatric surgery (Figure 2C). The mean ODIs before and after bariatric surgery were 39.5 ± 17.1 events/h and 10.9 ± 7.5 events/h, respectively.
iv) Impact of bariatric surgery on the mean oxygen saturation (mean SpO_2_)
Bariatric surgery was associated with a significant increase in the mean oxygen saturation [MD −1.94% (95%CI −2.5, −1.4)]. Out of the 30 studies, 10 included mean SpO_2_ before and after bariatric surgery (Figure 2D). The mean SpO_2_ levels before and after bariatric surgery were 92.3 ± 2.9% and 94.8 ± 1.3%, respectively.
v) Impact of bariatric surgery on the total sleep time spent with SpO_2_ < 90% (T-90)
Bariatric surgery was associated with a significant reduction in the total sleep time spent with SpO_2_ < 90% [MD 7.5 min (95%CI 5.0, 10.0)]. Out of the 30 studies, 7 included T-90 before and after bariatric surgery (Figure 2E). The mean T-90 values before and after bariatric surgery were 49.3 ± 69.1 min and 44.7 ± 76.8 min, respectively.
vi) Impact of bariatric surgery on the nadir oxygen saturation (L SpO_2_)
Bariatric surgery was associated with a significant increase in the nadir oxygen saturation [MD 9.0% (95%CI −11.8, −6.3)]. Out of the 30 studies, 13 included L SpO_2_ before and after bariatric surgery (Figure 2F). The mean L SpO_2_ values before and after bariatric surgery were 75.6 ± 4.7% and 84.6 ± 4.3%, respectively.

### 3.4. Sensitivity analysis

The heterogeneity (*I*^2^) values were high for all parameters obtained except T-90. Accordingly, a sensitivity analysis was conducted to assess potential sources of heterogeneity. Potential sources evaluated included the study design, the study population (i.e., the geographical location by continent), the mean age, the baseline BMI, the baseline AHI, the follow-up after surgery, the type of bariatric surgery, and the multiple use of the same study. A meta-regression analysis was conducted to reduce or resolve the heterogeneity.

i) BMI
The heterogeneity was high (*I*^2^ = 92.1%). After conducting a meta-regression analysis and adjusting for all potential sources of heterogeneity, *I*^2^ was reduced to 50.6% (factors that were significantly causing heterogeneity are the type of surgery-LSG, baseline BMI, mean age, and study design). Then, we used the trim-and-fill method and the leave-one study-out method. When we excluded the study of Wu et al. (2022), *I*^2^ was reduced to 16.5%, suggesting that this study was the main driving source for the heterogeneity.
ii) AHI
The heterogeneity was high (*I*^2^ = 95.5%). After conducting a meta-regression analysis and adjusting for all potential sources of heterogeneity, *I*^2^ was reduced to 47.7% (factors that were significantly causing heterogeneity are baseline BMI, baseline AHI, study design, and using the same study multiple times). Then, we used the trim-and-fill method and the leave-one study-out method. When we excluded the study of Wu et al. (2022), *I*^2^ was reduced to 2.5%, suggesting that this study was the main driving source for the heterogeneity.
iii) ODI
The heterogeneity was high (*I*^2^ = 88.5%). After conducting a meta-regression analysis and adjusting for all potential sources of heterogeneity, *I*^2^ was reduced to 54.0% (the factor that significantly caused heterogeneity was baseline AHI). Then, we used the trim-and-fill method and the leave-one study-out method. When we excluded the study of Shaarawy et al. (2016), *I*^2^ resolved suggesting that this study was the main driving source for the heterogeneity.
iv) Mean SpO_2_
The heterogeneity was high (*I*^2^ = 68.9%). After conducting a meta-regression analysis and adjusting for all potential sources of heterogeneity, *I*^2^ resolved (the factor that was driving heterogeneity was follow-up at 1 year).
v) T-90
There was no heterogeneity. Accordingly, no sensitivity analysis was conducted in this subgroup.
vi) L SpO_2_
The heterogeneity was high (*I*^2^ = 86.9%). After conducting a meta-regression analysis and adjusting for all potential sources of heterogeneity, *I*^2^ did not change appreciably (*I*^2^ = 86.7%). Then, we used the trim-and-fill method and the leave-one study-out method. When we excluded the study of Shaarawy et al. (2016), *I*^2^ was reduced to 13.6%, suggesting that this study was the main driving source for the heterogeneity. Of note, is that the type of surgery-LSG was also a significant factor contributing to the heterogeneity.

### 3.5. Publication bias

There was evidence of publication bias in the following parameters: BMI and AHI, ODI, and mean SpO_2_. This is most likely related to the following potential reasons. First, when we searched the databases we excluded non-English language articles, which can contribute to publication bias. Second, the funnel plots showed an asymmetrical distribution of the included studies across the midline, and finally, the Egger's test was statistically significant (BMI, *p* = 0.0001; AHI, *p* = 0.0008; ODI, *p* = 0.03; mean SpO_2_, *p* = 0.006), supporting the assumption that publication bias is very likely. There was no publication bias in T-90, *p* = 0.59 and L SpO_2_, *p* = 0.67. The funnel plots of these parameters are illustrated in Supplementary Figures S1A–F.

## 4. Discussion

The major findings from this systematic review and meta-analysis are as follows: (a) bariatric surgery (regardless of the type) is associated with a significant reduction in BMI; (b) bariatric surgery (regardless of the type) is associated with a significant reduction in AHI, which tends to be highest in patients with a high baseline AHI, high baseline BMI, and longer follow up; (c) bariatric surgery is associated with significant improvement in all other breathing-related PSG parameters (ODI, mean SpO_2_, T-90, and L SpO_2_).

Wong et al. (2018) conducted a systematic review and meta-analysis that focused on the impact of bariatric surgery on the AHI and to determine whether using different AASM hypopnea scoring roles (i.e., using the 3% or the 4% scoring role) can change the results. They included 27 studies in the qualitative analysis (24 non-RCTs and 3 RCTs) and 15 studies in the meta-analysis and concluded that bariatric surgery is more effective in reducing both AHI and BMI when compared to non-surgical weight loss strategies [WMD −25.1 events/h (95%CI −29.9, −20.2)] vs. [WMD −13.2 kg/m^2^ (95%CI −16.4, −10.0)]. They also found that higher baseline AHI and BMI, as well as a longer duration of follow-up, were associated with greater reductions in weight and AHI. There was no association between the amount of weight loss and the reduction in AHI. After 1 year, Zhang et al. (2019) published a systematic review and meta-analysis that focused on the impact of bariatric surgery on sleep-related hypoxemia. They included 15 studies (14 non-RCTs and 1 RCT) and concluded that bariatric surgery resulted in a significant improvement in nocturnal hypoxia. Mean SpO_2_ increased by 1.36% {[95% CI (0.72, 2.00)], *p* < 0.001} at a mean of 12.5 months, and the nadir SpO_2_ increased by 1.08% {[95% CI (0.68, 1.49)], *p* < 0.001} at a mean of 10.1 months. Furthermore, their review showed a significant reduction in both AHI and BMI with bariatric surgery. The results of our systematic review and meta-analysis are updates of both reviews (2019–2023) and still support the significant positive impact of bariatric surgery on both obstructive sleep apnea and sleep-related hypoxia.

Although we excluded studies that used PAP therapy after bariatric surgery (a total of 18 studies), we cannot ignore the impact of bariatric surgery on weight loss and the subsequent OSA severity in these studies. In fact, the BMI and AHI were significantly reduced in all these studies. The mean BMIs pre- and post-bariatric surgery were 48.2 ± 6.7 kg/m^2^ and 36.2 ± 5.1 kg/m^2^, respectively. The mean AHIs pre- and post-bariatric surgery were 49.1 ± 20.9 events/h and 18.8 ± 13.4 events/h, respectively over a mean follow-up of 12.7 months (data not included).

As mentioned earlier, there is a correlation between OSA and several co-morbid diseases, especially cardiovascular diseases. The prevalence of OSA and the impact of treating OSA in cardiovascular diseases has been studied extensively. OSA is highly prevalent in patients with hypertension, and up to 80% of patients with treatment-resistant hypertension have OSA (Logan et al., 2001). OSA treatment has been shown to reduce blood pressure, although this reduction was only 2–3 mmHg (Fava et al., 2014). Similarly, OSA is an independent risk factor for atrial fibrillation in patients without underlying cardiovascular diseases (Mehra et al., 2006). Several small retrospective studies have shown that OSA treatment can reduce the atrial fibrillation burden independent of the modality of rhythm control (Patel et al., 2017). Similarly, OSA treatment has been shown to be promising in patients with pulmonary hypertension and cerebrovascular diseases (Sajkov et al., 2002; Brill et al., 2018). The American Heart Association recently published a scientific statement that recommends screening for sleep-related breathing disorders in patients with poorly controlled/treatment-resistant hypertension, recurrent atrial fibrillation, New York Heart Association class II–IV HF and suspicion of sleep-disordered breathing, tachy-brady syndrome, ventricular tachycardia, survivors of sudden cardiac death in whom sleep apnea is suspected, and stroke (Yeghiazarians et al., 2021).

Conservative lifestyle interventions (such as diet and medications) are effective tools for weight loss and AHI reduction (Blackman et al., 2016; Carneiro-Barrera et al., 2022). However, bariatric surgery seems to be more effective than conservative interventions in treating OSA. Dixon et al. conducted a randomized clinical trial and found that patients in the conventional weight loss group lost a mean of 5.1 kg (95% CI, 0.8–9.3 kg) compared to 27.8 kg (95% CI, 20.9–34.7 kg) in the bariatric surgery group (*P* < 0.001). The AHI decreased by 14.0 events/h (95% CI, 3.3–24.6 events/h) in the conventional weight loss group and by 25.5 events/h (95% CI, 14.2–36.7 events/h) in the bariatric surgery group (Dixon et al., 2012).

The strengths of our systematic review and meta-analysis are the following:

- a) To our knowledge, it includes the largest number of studies documenting the effects of bariatric surgery on obstructive sleep apnea.
- b) To study the impact of bariatric surgery on OSA in the absence of other factors, we excluded studies that used PAP therapy after bariatric surgery.
- c) We did not rely on AHI as a surrogate for OSA. Rather, we assessed all breathing-related PSG parameters.

Our review has two major limitations. First, since we excluded many studies that used PAP therapy after bariatric surgeries, many excellent studies (including RCTs) were excluded. Second, the heterogeneity was high for all parameters except T-90. However, we were able to conduct a sensitivity analysis and identify the potential sources of heterogeneity.

## 5. Conclusions

This systematic review and meta-analysis concluded that bariatric surgery reduces the severity of obstructive sleep apnea and affects several sleep-related breathing parameters. Patients with sleep-related breathing disorders and morbid obesity who are at high risk of cardio-metabolic diseases and failing conservative lifestyle interventions should be evaluated for bariatric surgery. Further research is warranted to reveal more facts about the correlation between weight loss and airway dynamics and to determine how that can help with OSA treatment.

## Data availability statement

The original contributions presented in the study are included in the article/Supplementary material, further inquiries can be directed to the corresponding author.

## Author contributions

SM contributed to the conception, design of the study, and wrote the first draft of the manuscript. AR, PR, YA, DC, MK, KH, SH, SP, and IG organized the database and reviewed the manuscript. PR performed the statistical analysis. PR and AR wrote sections of the manuscript. SP and IG mentored and supervised the research activity. All authors contributed to the manuscript revision, read, and approved the submitted version of the manuscript.
