# Supplementary material for: The impact of bariatric surgery on breathing-related polysomnography parameters—Updated systematic review and meta-analysis
Source: Front Sleep. 2023 Aug 3;2:1212936. doi: 10.3389/frsle.2023.1212936 (PMC12713882; doi:10.3389/frsle.2023.1212936)
Supplement: Supplementary file 1 [file Data_Sheet_1.docx]

***Supplementary Data***

**The Impact of Bariatric Surgery on Breathing-Related Polysomnography Parameters – Updated Systematic Review and Meta-analysis**

**Saif Mashaqi1,2*, Anas Rihawi3, Pooja Rangan5,6, Katherine Ho4, Mateen Khokhar7, Sonia Helmick8, Yazan Ashouri9, Daniel Combs2, Iman Ghaderi10, Sairam Parthasarathy1,2**
1Department of Pulmonary, Critical Care, Allergy and Sleep Medicine, University of Arizona College of Medicine, Tucson, AZ, USA

2University of Arizona Health Sciences Center for Sleep & Circadian Sciences, Tucson, AZ, USA

3Department of Pulmonary and Critical Care medicine, Oregon Health and Science University (OHSU), Portland, OR

4Department of General Surgery, Johns Hopkins University School of Medicine, Baltimore, MD

5Banner University Medical Center Phoenix. Department of Medicine. Phoenix, AZ

6The University of Arizona College of Medicine Phoenix. Department of Internal Medicine. Phoenix, AZ

7Department of Internal Medicine, Dignity Health Medical Group St Joseph’s, Phoenix, AZ

8Department of Pulmonary and Sleep medicine, Tucson VA Medical Center. Tucson, AZ

9Department of Neurology, St. Vincent Medical Center, Toledo, OH

10Section of Minimally Invasive, Robotic and Bariatric Surgery, Department of Surgery, University of Arizona College of Medicine, Tucson, AZ, USA

*** Correspondence:**

Saif Mashaqi, MD

The University of Arizona / Banner University Medical Center

1625 N Campbell Ave, Tucson, AZ 85719

[saifmashaqi@arizona.edu](mailto:saifmashaqi@arizona.edu)

1. **Supplementary Figures and Tables**

| Criteria | Yes | No | Other (CD, NR, NA)* |
| --- | --- | --- | --- |
| 1. Was the research question or objective in this paper clearly stated? |  |  |  |
| 2. Was the study population clearly specified and defined? |  |  |  |
| 3. Was the participation rate of eligible persons at least 50%? |  |  |  |
| 4. Were all the subjects selected or recruited from the same or similar populations (including the same time period)? Were inclusion and exclusion criteria for being in the study prespecified and applied uniformly to all participants? |  |  |  |
| 5. Was a sample size justification, power description, or variance and effect estimates provided? |  |  |  |
| 6. For the analyses in this paper, were the exposure(s) of interest measured prior to the outcome(s) being measured? |  |  |  |
| 7. Was the timeframe sufficient so that one could reasonably expect to see an association between exposure and outcome if it existed? |  |  |  |
| 8. For exposures that can vary in amount or level, did the study examine different levels of the exposure as related to the outcome (e.g., categories of exposure, or exposure measured as continuous variable)? |  |  |  |
| 9. Were the exposure measures (independent variables) clearly defined, valid, reliable, and implemented consistently across all study participants? |  |  |  |
| 10. Was the exposure(s) assessed more than once over time? |  |  |  |
| 11. Were the outcome measures (dependent variables) clearly defined, valid, reliable, and implemented consistently across all study participants? |  |  |  |
| 12. Were the outcome assessors blinded to the exposure status of participants? |  |  |  |
| 13. Was loss to follow-up after baseline 20% or less? |  |  |  |
| 14. Were key potential confounding variables measured and adjusted statistically for their impact on the relationship between exposure(s) and outcome(s)? |  |  |  |

**Table S1.** Quality assessment tool sample for non-RCTs developed by the National Heart, Lung, and Blood Institute

| **Study** | **General rating** | **Study notes** |
| --- | --- | --- |
| Kara et al. 2021 | Good |  |
| Valencia-Flores et al. 2004 | Fair | -High number of losses to follow-up participants.  -Not all eligible participants were enrolled in the study. |
| Zou et al. 2015 | Good |  |
| Pillar et al. 1994 | Fair | -Study question, I/E criteria not precisely defined |
| Bae et al. 2014 | Good |  |
| Tirado et al. 2017 | Good |  |
| Nitipatana Chierakul et al. 2020 | Good |  |
| Peromaa-Haavisto et al.  2016 | Good |  |
| Shaarawy et al. 2016 | Fair | -Small sample size  -Not blinded |
| Song et al. 2021 | Fair | -Small sample size  -Not blinded |
| Al-Jumaily et al. 2018 | Fair | -Small sample size  -Not blinded |
| Busetto et al. 2004 | Fair | - Small sample size.  - Didn’t state how the patients were selected. |
| Peiser et al. 1983 | Fair | -Only apnea-index, no LSaO2,  -Data reporting was not very clear |
| Lage-Hansen et al. 2018 | Good |  |
| Jiao et al. 2016 | Fair | -Small sample size, selection bias (all participants are diabetic) |
| Genio et al. 2016 | Fair | -Small sample size |
| Fredheim et al. 2013 | Good |  |
| Bakker et al. 2013 | Good |  |
| Bakker et al. 2014 | Good |  |
| Fritscher et al. 2007 | Fair | -Selection bias  -Not all eligible participants were enrolled in the study. |
| Fatmanur Karaköse et al.  2014 | Fair | -Not all control patients undergo PSG. |
| Kreigar et al. 2012 | Good |  |
| Pallayova et al. 2011 | Good |  |
| Morong et al. 2013 | Good |  |
| Xu et al. 2021 | Fair | -Study objectives were not clearly stated  - I/E criteria were not clearly identified.  -Drop out. |
| Wu et al. 2022 | Good |  |
| Yanari et al. 2022 | Good |  |
| Obeidat et al. 2020 | Fair | -The study objectives were not clear  -I/E criteria were not precise and detailed  -Not clear the dropout rate |

**Table S2**. Quality assessment results for non-RCTs developed by the National Heart, Lung, and Blood Institute

| **Domain** | **Low risk** | **Some concern** | **High risk** |
| --- | --- | --- | --- |
| Bias arising from the randomization process |  |  |  |
| Bias due to deviations from intended interventions |  |  |  |
| Bias due to missing outcome data |  |  |  |
| Bias in measurement of the outcome |  |  |  |
| Bias in selection of the reported result |  |  |  |
| Overall bias |  |  |  |

**Table S3**. Cochrane Risk of Bias (ROBINS2) assessment tool

| **Studies/Domains** | **Aguiar et al 2014** | **Bakker et al 2018** |
| --- | --- | --- |
| Randomization process | Low | Low |
| Deviations from intended | Low | Some concerns |
| Missing outcome data | Low | Low |
| Measurement of the outcome | Low | Some concerns |
| Selection of the reported results | Low | Low |
| Overall bias | Low | Some concerns |

**Table S4**. Quality assessment results for RCTs developed by Cochrane Risk of Bias (ROBINS2) assessment tool.

| **GRADE worksheet** | **Rating** | **Study notes** | **Quality of the evidence** |
| --- | --- | --- | --- |
| Primary outcome (AHI and BMI)  Secondary outcome (ODI, mean SpO2, T-90, L SpO2) | | | |
| **Risk of bias** | Very serious (-2) | -Most studies are before and after bariatric surgery.  -Some studies included only males or females patients.  -High dropout rate.  -CPAP was used in some patients after bariatric surgery (overlap). | Very low |
| **Inconsistency** | No | -All studies had relevant populations and intervention.  -All studies had consistent outcome and treatment effect (i.e., reduction in BMI, AHI, ODI, T-90 and increase in mean SpO2, L SpO2)  On the other hand  -Variable follow up times (ranging from a month to up to 5 years).  -High heterogeneity (I2). |  |
| **Indirectness** | No | -The outcome was clearly addressed in all studies. |  |
| **Imprecision** | No | -Confidence interval did not cross zero.  -Intervention was beneficial.  - variable sample sizes (n=10 to 162) |  |
| **Publication Bias** | Yes (-1) | -Funnel plots (asymmetrical distribution)  -Egger’s test |  |
| **Large effect** | Large (+1) | -Despite study designs, there was a significant change in all primary and secondary outcomes. |  |

**Table S5.** Certainty of evidence results using the GRADE worksheet.


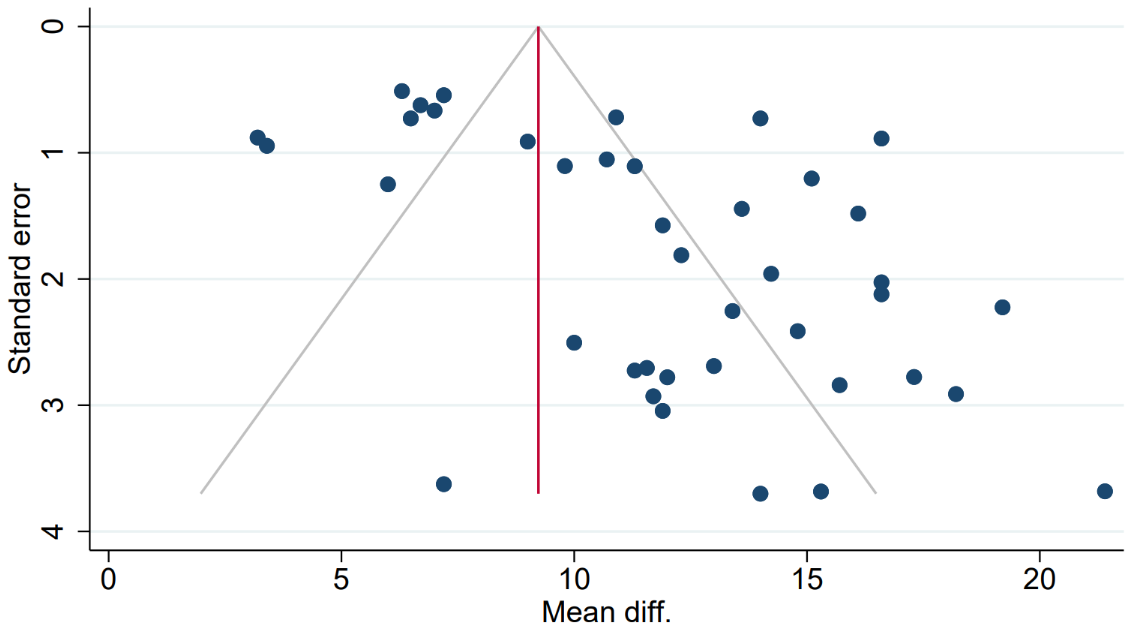

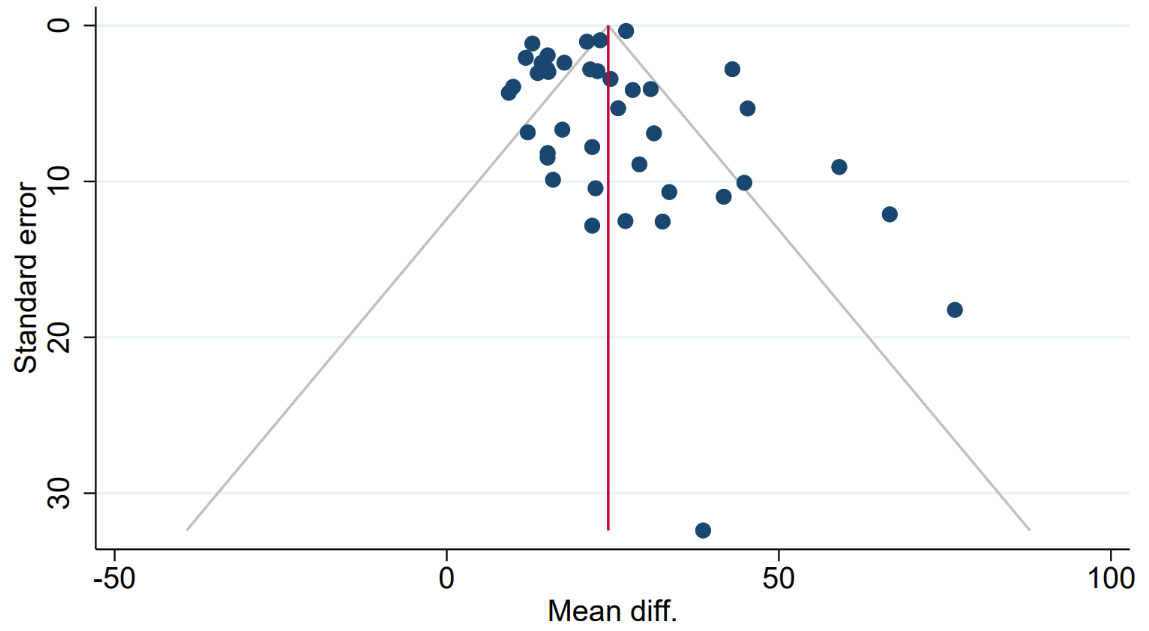

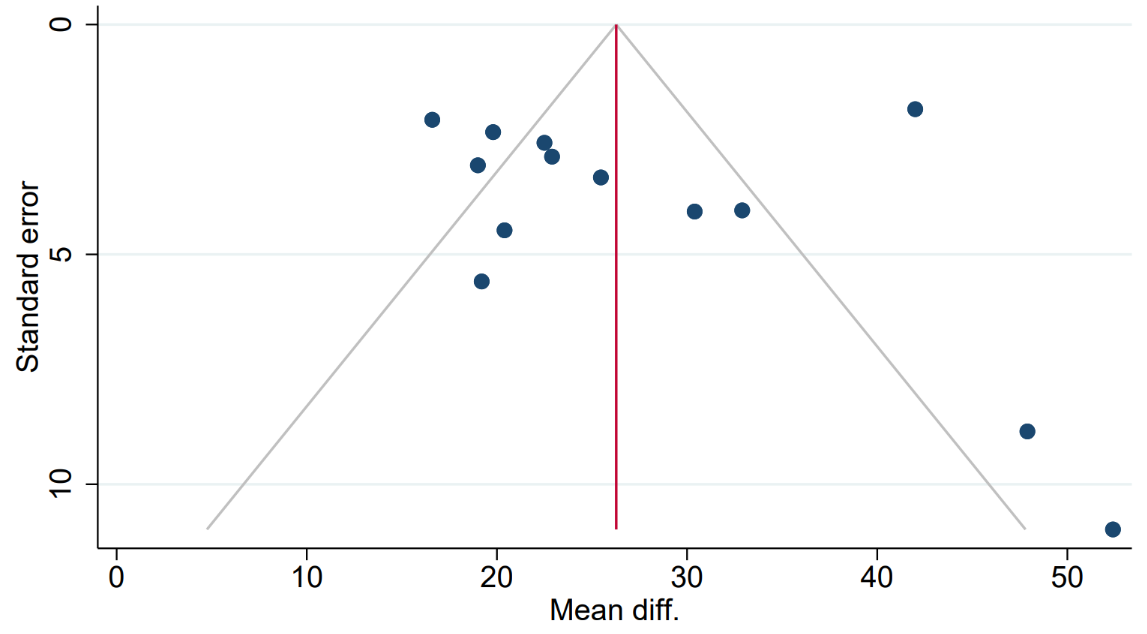


A) BMI B) AHI C) ODI


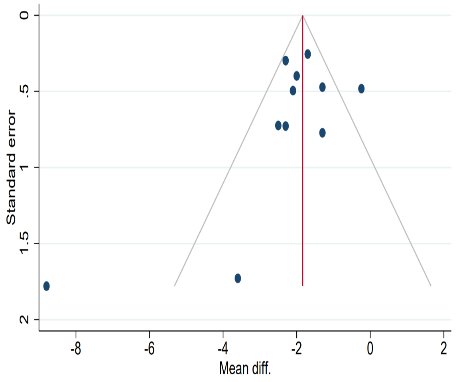

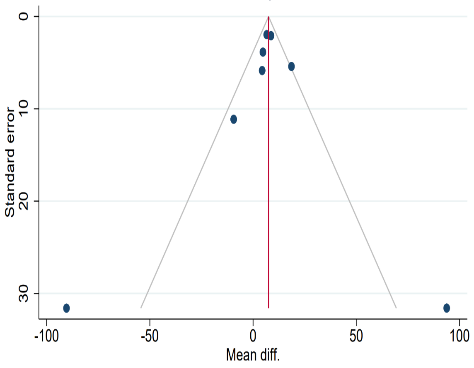

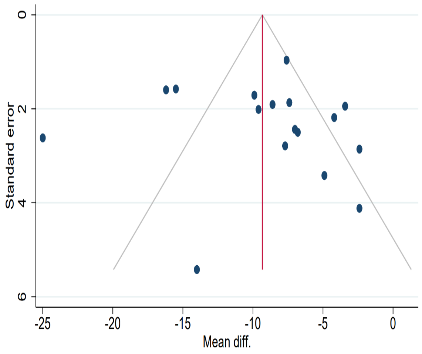


D) Mean SpO2 E) T-90 F) L SpO2

**Figure S1.** Publication bias Funnel plots for all breathing-related PSG parameters and BMI.
